# Thoracic Epidural analgesia versus Rectus Sheath Catheters for open midline incisions in major abdominal surgery within an enhanced recovery programme (TERSC): study protocol for a randomised controlled trial

**DOI:** 10.1186/1745-6215-15-400

**Published:** 2014-10-21

**Authors:** Kate M Wilkinson, Anton Krige, Sarah G Brearley, Steven Lane, Michael Scott, Anthony C Gordon, Gordon L Carlson

**Affiliations:** Department of Anaesthesia and Critical Care, Royal Blackburn Hospital, Blackburn, BB2 3HH UK; Faculty of Health and Medicine, Division of Health Research, Lancaster University, Lancaster, LA1 4YT UK; Department of Biostatistics, University of Liverpool, Duncan Building, Liverpool, L69 3GA UK; Department of Anaesthesia and Intensive Care, Royal Surrey County Hospital, University of Surrey, Guildford, GU2 7XX UK; Section of Anaesthetics, Pain Medicine and Intensive Care, Department of Surgery and Cancer, Imperial College, London, SW7 2AZ UK; Department of Surgery, Salford Royal NHS Foundation Trust, Stott Lane, Salford, M6 8HD UK

**Keywords:** Rectus sheath catheters, Rectus sheath block, Analgesia, Epidural, Enhanced recovery, Laparotomy

## Abstract

**Background:**

Thoracic epidural analgesia (TEA) is recommended for post-operative pain relief in patients undergoing major abdominal surgery via a midline incision. However, the effectiveness of TEA is variable with high failure rates reported post-operatively. Common side effects such as low blood pressure and motor block can reduce mobility and hinder recovery, and a number of rare but serious complications can also occur following their use.

Rectus sheath catheters (RSC) may provide a novel alternative approach to somatic analgesia without the associated adverse effects of TEA. The aim of this study is to compare the efficacy of both techniques in terms of pain relief, patient experience, post-operative functional recovery, safety and cost-effectiveness.

**Methods/design:**

This is a single-centre randomised controlled non-blinded trial, which also includes a nested qualitative study. Over a two-year period, 132 patients undergoing major abdominal surgery via a midline incision will be randomised to receive either TEA or RSC for post-operative analgesia. The primary outcome measures pain scores on moving from a supine to a sitting position at 24 hours post wound closure, and the patient experience between groups evaluated through in-depth interviews. Secondary outcomes include pain scores at rest and on movement at other time points, opiate consumption, functional recovery, morbidity and cost-effectiveness.

**Discussion:**

This will be the first randomised controlled trial comparing thoracic epidurals to ultrasound-guided rectus sheath catheters in adults undergoing elective midline laparotomy. The standardised care provided by an Enhanced Recovery Programme makes this a comparison between two complex pain packages and not simply two analgesic techniques, in order to ascertain if RSC is a viable alternative to TEA.

**Trial registration:**

Current Controlled Trials ISRCTN81223298 (16 January 2014).

**Electronic supplementary material:**

The online version of this article (doi:10.1186/1745-6215-15-400) contains supplementary material, which is available to authorized users.

## Background

### Background and rationale

Enhanced Recovery Programmes (ERP) consist of a series of protocolised multimodal interventions aimed at reducing complications, thereby expediting recovery [1]. One of the key elements in all ERP is the provision of adequate post-operative analgesia. This reduces the stress response, improves patient wellbeing and allows early mobilisation. Early mobilisation itself is important to reduce secondary complications such as chest infection and deep vein thrombosis. Post-operative insulin resistance, which has been linked to increased complications, can be reduced by muscle activity [1]. Thoracic epidural analgesia (TEA), which facilitates dynamic analgesia and early mobilisation, is currently the standard for post-operative analgesia following major abdominal surgery within ERP [1].

A number of systematic reviews and meta-analyses have demonstrated that TEA is associated with superior post-operative analgesia and fewer adverse events when compared to high dose systemic opiate administration [2–8]. High dose systemic opiates are strongly associated with post-operative sedation, ileus [8–11] and nausea and vomiting, all of which impair post-operative recovery and prolong hospital stay. Ileus is the most common complication following abdominal surgery, and is responsible for significant prolongation of recovery times. TEA may reduce ileus rates as compared to opiates [12–17].

TEA is occasionally contraindicated and may also lead to rare but significant complications, for example nerve injury, epidural haematoma and epidural abscess [18]. More common adverse effects of TEA include motor blockade of the lower limbs and urinary retention requiring catheterisation, both of which can impede post-operative mobilisation and recovery. Additionally, the need to use epidural opioids to prevent the sensory regression associated with the use of local anaesthetics alone may contribute in itself to an increase in post-operative ileus [13–15]. Hypotension, resulting from sympathetic nervous blockade, is common with TEA and may result in iatrogenic fluid overload [19]. Fluid overload is also associated with ileus and other post-operative complications, including anastomotic dehiscence, and has been shown to independently predict increased length of hospital stay [20]. TEA has also been found to have a high reported failure rate (25 to 30%) [2–8]. These failures are multifactorial and usually managed either by epidural replacement, substitution with systemic opiate infusion or both [21, 22].

Rectus sheath catheters (RSC) are a regional anaesthetic technique in which the ventral rami of the seventh to twelfth intercostal nerves, which supply the rectus abdominis muscle and overlying skin, are blocked [23, 24]. This provides midline somatic analgesia, with additional doses of systemic opiate required for visceral analgesia (12 to 36 hours duration) [23, 24]. This sensory blockade is achieved by injecting local anaesthetic into the potential space between the rectus muscle and the posterior rectus sheath. As a single bolus of local anaesthetic has a maximum duration of 12 hours [23, 24] it is necessary to insert a catheter into this space to allow either a continuous infusion of local anaesthetic, or repeated boluses of local anaesthetic every 8 to 12 hours for 48 to 72 hours post-operatively. Although this regional block was first described at the turn of the last century [23, 24], it was used infrequently until the recent availability of long-acting local anaesthetic agents, small portable ultrasound machines [25] and small calibre infusion catheters. These developments have resulted in renewed interest in RSC for the management of post-operative pain following midline abdominal incisions [23, 24, 26–29].

RSC offer several potential advantages over TEA, which may make them ideally suited for use in an ERP. In particular, they avoid hypotension and motor blockade. RSC are also inserted after the induction of general anaesthesia, which patients may prefer to the usual awake insertion of epidurals. No complications or serious adverse events related to RSC have been reported to date [23, 24], but local anaesthetic toxicity [30–33] (which applies to all regional anaesthetic techniques including epidurals) and visceral injury during insertion are hypothetical possibilities.

The existing literature relating to the use of RSC is limited, with published studies all utilising surgically inserted RSC to compare local anaesthetic with saline [34–40] or non-randomised trials comparing RSC to epidurals [38, 40]. Despite this, the majority of studies demonstrate proof of concept [34–37, 39]. This randomised controlled trial will compare ultrasound-guided insertion of RSC with TEA for midline laparotomy incisions, in adults undergoing elective major abdominal surgery within an ERP pathway.

### Objectives

The aim of the study is to assess the efficacy, safety and acceptability to patients of RSC. To this end, a qualitative study has been nested within the primary study design to facilitate understanding of the patient experience.

### Trial design

This is a randomised parallel group concealed allocation non-blinded superiority trial with a nested qualitative study of a subset of patients.

## Methods: participants, interventions and outcomes

### Study setting

TERSC is a single-centre trial, which will take place at the Royal Blackburn Hospital (RBH), a large district general hospital in England providing services to a local catchment population of 530,000 and complex regional services to a population of 2 million.

### Eligibility criteria

All adult (18 or over) patients who are listed for elective major abdominal surgery via a midline incision, and who meet all inclusion and exclusion criteria (see Table 1), will be eligible for recruitment into the trial.

**Table 1:** **Inclusion and exclusion criteria**

Inclusion criteria	Exclusion criteria
• Patients >18 years of age	• Contraindication to epidural analgesia: for example, coagulopathy, local infection, systemic sepsis, severe aortic stenosis
• Planned major abdominal surgery including major colorectal resections, pancreaticoduodenectomy and radical cystectomy	• Consent refused for either TEA or RSC
• Planned open midline surgical incision	• Non-English speaker
• Included in the ERP	• Ano-rectal excision: for example, pan-proctocolectomy or abdomino-perineal resection.
• Willing and able to give consent	• Planned transverse or oblique incisional approach
• ASA (American Society of Anesthesiologists) 1 to 3	• Allergy to local anaesthetic drugs or opiates
	• Opiate tolerance
	• Pre-existing chronic abdominal pain
	• Extensive existing midline abdominal scarring

Abbreviations: ERP, Enhanced Recovery Programme; RSC, rectus sheath catheter; TEA, thoracic epidural analgesia.

### Interventions

#### Standard general anaesthetic

All patients in the trial will receive a general anaesthetic as follows: propofol 2 to 4 mg/kg intravenously (IV); remifentanil 1 mcg/kg or target controlled infusion (effect site target-Minto model) or fentanyl 1 to 2 mcg/kg IV; atracurium 0.5 mg/kg or rocuronium 0.5 mg/kg. Anaesthesia will be maintained as follows: isoflurane, sevoflurane or desflurane, initially at 1 MAC (minimum alveolar concentration) then titrated to clinical end points; remifentanil (RSC arm only) 0.1 to 0.5 mcg/kg/min or target controlled infusion (effect site target-Minto model) titrated to clinical effect; oxygen in air titrated to SaO2 > 95%; and multimodal anti-emesis consisting of ondansetron 4 to 8 mg, dexamethasone 8 mg and cyclizine 50 mg.

Maintenance fluid will consist of Plasma-Lyte B (Baxter, Compton, Newbury, Berkshire, UK) at 2 to 4 mls/kg/hr, and goal-directed fluid therapy utilising 250 ml fluid boluses of Plasma-Lyte B until stroke volume is maximised. This will be guided by either oesophageal Doppler or Edwards Flotrac (Edwards Lifesciences, Irvin, California, USA) cardiac output monitoring.

#### Rectus sheath catheters

The RSC will be inserted bilaterally under ultrasound guidance (see Additional file 1 for description of the technique) [23–27] by the anaesthetist immediately following induction of a standard general anaesthetic. Only anaesthetists who have successfully inserted RSC under the supervision of the chief investigator (CI) will undertake the insertion of RSC. An aseptic technique will be used and 20 ml of 0.25% bupivacaine will be injected via each catheter into the potential space between the rectus muscle and the posterior rectus sheath, 2 to 4 cm either side of the midline. The catheters will be tunnelled subcutaneously to a level above the costal margin.

Approximately 45 minutes before the end of surgery a 10-mg bolus of IV morphine will be administered to provide visceral analgesia. A transdermal fentanyl patch (12 mcg if ≥70 years and/or ≤65 kg and 25 mcg if <70 years and >65 kg; 72-hour duration of action) will be applied, and if more than 3 hours have passed since the initial RSC bolus, a further 40 ml of 0.2% ropivacaine will be administered via an ambIT^R^ (Summit Medical Products Inc, South Sandy, Utah, USA) Preset PCA or ambIT^R^ mini infusion pump (split between the 2 RSC via a Y-connector). The pumps will be set to deliver boluses of 40 mls 0.2% ropivacaine, with a 4-hour lockout, each time the bolus button is pressed [30–33]. The bolus button may be staff- or patient-activated but will be pressed regularly every 4 hours post-operatively. Each 40 ml bolus takes 24 minutes to deliver; therefore the total time from the start of one bolus to the start of the next will be 4 hours and 24 minutes.

If breakthrough pain is experienced, staff will first ensure that the RSC have been utilised as per protocol. If more than 4 hours have elapsed since the last bolus of local anaesthetic then 40 mls of 0.2% ropivacaine will be administered via the pump. If inadequate pain relief follows the bolus, or if no bolus is due, then 10 mg oral morphine or 5 mg oral oxycodone will be used as required for further breakthrough pain within the first 48 hours.

#### Thoracic epidural analgesia

Epidurals will be sited prior to the induction of general anaesthesia at T7 to T9 for a right-sided colonic resection or upper gastrointestinal (GI) surgery, and T9 to T11 for a left-sided resection or radical cystectomy. Standard aseptic insertion technique will be followed, using loss of resistance to air or saline as per preference. Following a suitable test dose, a bolus of 10 mls 0.25% bupivacaine with 100 mcg fentanyl will be administered to establish a block.

Following insertion, an epidural infusion of 0.125% bupivacaine and 2 mcg/ml fentanyl will be commenced at 10 mls/hour and then titrated to effect. On the second post-operative night, a fentanyl patch (12 mcg if ≥70 years and/or ≤65 kg and 25 mcg if <70 years and >65 kg) will be applied after which the epidural will be weaned overnight and removed the following morning as per local clinical protocol.

If breakthrough pain is experienced, the epidural block will first be optimised with an additional bolus of 10 to 20 mls of the maintenance solution. If inadequate pain relief follows the bolus then 10 mg oral morphine or 5 mg oral oxycodone will be used as required for further breakthrough pain within the first 48 hours.

All reasonable attempts will be made to rescue failing study interventions. These may include delivering an additional bolus of local anaesthetic, altering the catheter position, correcting blood pressure to allow continuation of the study intervention, and (in the case of TEA) repositioning the patient. However, if complete failure of the study intervention occurs within the first 48 hours post-operatively, it will be replaced by IV morphine patient controlled analgesia (PCA) following an adequate IV bolus of morphine. Complete failure is defined as complete lack of any sensory blockade and any improvement in the severity of pain reported by the patient following an adequate bolus injection of local anaesthetic via the TEA or RSC.

Patients in both arms of the trial will receive all other hospital care according to our institution’s ERP. Pre-operatively, this consists of pre-operative assessment, planning and preparation (including cardiopulmonary exercise testing if aged ≥60 years or <60 years with cardiorespiratory comorbidity), ERP information, a patient diary and PreLoad (Vitaflo International Limited, Liverpool, UK) drinks (sachets of maltodextrin powder for reconstitution in 500 mls water; 2 to be consumed the night before and an additional one 2 to 3 hours prior to surgery). Upper GI and right-sided colonic resections receive no bowel preparation and left-sided colonic resections, rectal resections and radical cystectomies will receive phosphate enemas on the morning of surgery. All patients receive 1 g paracetamol and 300 mg gabapentin orally pre-operatively.

Intra-operatively, in addition to the standard general anaesthetic and interventions already described, nasogastric tubes are avoided, surgical drains minimised and normothermia is maintained using enFlow® (GE Healthcare, Little Chalford, Buckinghamshire, UK) IV fluid warmers and Bair Hugger™ (3 M, St Paul, Minnesota, USA) external warming blankets.

Post-operatively, in addition to the randomised primary analgesia technique all patients receive multimodal oral analgesia, comprising 1 g paracetamol 6-hourly orally or IV if not tolerating oral; 400 mg ibuprofen 8-hourly orally once tolerating full oral diet; and 100 mg gabapentin 8-hourly orally (for 72 hours). A transdermal fentanyl patch is applied with dose and timing relevant to the randomised intervention as described above. Post-operative nausea and vomiting is minimised with 3 mg Buccastem 8-hourly buccally for 48 hours. Patients will undergo structured mobilisation from day 1 post-operatively. IV fluids are minimised and 2 litres per day of oral fluid is encouraged with full oral diet encouraged from day 1 post-operatively. Urinary catheters will be removed on day 1 in the RSC group and on removal of the epidural in the TEA group.

#### Qualitative study

A cross-sectional study will achieve the aim to study the patient experience of both interventions. This will utilise an inductive approach informed by the principles of grounded theory [41]. It uses maximum variety sampling to enable a range of experiences to be sought rather than seeking to make generalisations based on population characteristics. The anticipated sample size is 10 per trial arm (20 in total) and interviews will be undertaken by an experienced researcher at a place convenient to the participant (usually their home). During the qualitative phase all consenting RCT trial participants will be invited to take part in the qualitative study RCT (randomised controlled trial). They will be contacted again 2 to 3 weeks after the intervention, invited to participate and re-consented to avoid any issue of coercion whereby patients feel that they have to consent to the qualitative study in order to gain entry onto the RCT.

Face-to-face interviews will then take place 4 weeks post-intervention. This time point has been chosen to avoid the initial recovery period after hospital discharge, whilst still allowing the generation of rich data concerning the experience of RSC or TEA (we expect most patients to be discharged between 8 and 14 days, and to require a 3-month leave of absence from work). Data will be collected through in-depth interviews focusing on the participant’s expectations, experiences and outcomes. The interviews will be digitally audio recorded and fully transcribed (specifically covered in the consent process). Constant comparative thematic analysis will be undertaken to detect commonalities and differences within the accounts and across the two groups. Constant comparison permits the early stages of analysis to identify emergent issues and inform later data collection in order to produce insights grounded in the experiences of the participants.

#### Outcomes

The primary outcome measure of the RCT is the difference in mean pain score on movement from supine to sitting position at 24 hours after extubation between groups, measured using a Visual Analogue Scale (VAS) of 0 to 100 mm with 0 being no pain and 100 mm being the worst pain imaginable [42, 43]. VAS is a standard tool to compare analgesic efficacy between interventions in pain studies, and pain control adequate to allow dynamic movement at 24 hours is considered key to successful recovery after major surgery, with full mobilisation thus potentially feasible. An additional primary outcome measure is the difference in patient experience measured by the nested qualitative study utilising interviews conducted one month after surgery in a subset of patients from the main trial.

Secondary outcomes include further comparisons of analgesic efficacy and effectiveness, functional recovery, safety and cost-effectiveness. Further analgesic quality measurements include mean VAS pain scores at rest and on movement at 2, 6, 12, 24, 48 and 72 hours after extubation between intervention groups to compare the efficacy of analgesia at different clinically relevant time points and the time-based evolution of analgesic efficacy; the difference in sleep quality measured by VAS scores on the first three mornings post-operatively; the difference in self-assessed functional pain categorising respiratory function and mobility measured between 4 pm and 5 pm on each of the first 3 post-operative days; time to first rescue opiate; the total opiate consumption (excluding the protocolised fentanyl) over the first 48 hours post-operatively; and an overall score of satisfaction with pain relief on day 3 post-operatively.

Functional recovery will be compared assessing the difference in decrement of the Post-operative Quality Recovery Scale (PQRS) score [44] between groups, from baseline measurement to the measurements obtained on days 4, 7 and 30 post-operatively using a validated questionnaire [44]; time to first mobilisation; the difference in time to return of GI function; achievement of the daily ERP mobilisation goals (for example, 4 × 60 m walks on day 2 post-operatively); adherence to the local ERP pathway; time until patients meet standardised clinical discharge criteria as per the hospital ERP [45, 46]; and the actual length of hospital stay. Return of GI function is defined as the time to tolerance of full diet, passage of flatus and bowel opening. Tolerance of a full diet will occur when the patient has consumed more than 90% of the planned intake of a meal, and does not feel nauseated or vomit after any meal. If four meals are consumed and the fourth vomited, then time to tolerating a full meal will not have been reached.

Safety measurements are differences in fluid balance and the incidence of hypotension over the first 48 hours post-operatively. Overall fluid balance will be measured using the fluid balance charts and the difference between pre-operative body weight and body weight measured on the first three mornings post-operatively (positive fluid balance is strongly correlated with morbidity in this cohort and may be linked to side effects of the trial interventions, that is hypotension). Any episode of hypotension, defined as a systolic blood pressure less than 90 mmHg, a mean arterial pressure less than 65 mmHg, or a reduction of greater than 20% from the pre-operative blood pressure, will be noted. Vasoconstrictor usage will be measured as a surrogate of haemodynamic stability.

Other measures of morbidity include nausea and vomiting measured by a VAS score, the incidence of ileus and the POMS (Post-Operative Morbidity Score). Ileus has been defined as new nausea and vomiting, abdominal distension, and abdominal discomfort with loss of bowel sounds [47]. The POMS is a score of post-operative morbidity validated in the UK which applies a binary outcome to each of 9 domains on either day 3, 5 or 7 post-operatively, chosen depending on the type of surgery [48]. Day 5 will be used for this study. Any surgical complications occurring within 30 days post-operatively will be classified for severity using the Clavien-Dindo classification [49].

If a study intervention is deemed to fail, then the timing of the failure, any replacement technique and the reason will be recorded: for example, block failure (pain and no demonstrable block of abdominal dermatomes); catheter disconnection; excessive catheter leakage; premature catheter removal; cessation of infusion due to hypotension; respiratory depression or possible local anaesthetic toxicity (respiratory rate <8/minute or the need for naloxone, systolic BP <80 or MAP <50; pruritus; circumoral paraesthesia; seizures; obtundation; arrhythmias; and cardiovascular collapse).

A full health economic assessment calculating Quality Adjusted Life Years is beyond the scope of this study. However, short-term cost-effectiveness will be calculated, taking into account the cost of consumables, insertion and management time for the 2 techniques, the respective length of stay both in hospital and critical care, and readmissions or re-operations within 30 days of surgery [50].

#### Participant timelines

**Figure 1:**
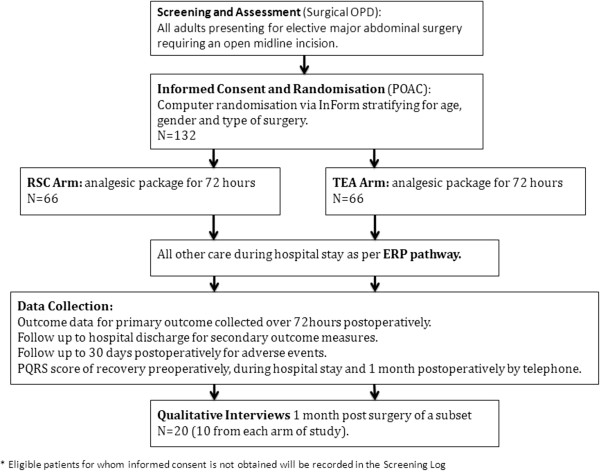
**Trial schema.** Diagram illustrating timelines and patient pathways for both study arms.

When patients are informed of the need for an operation by their surgical team, verbal agreement will be ascertained for their details to be forwarded to the trial team, enabling the trial nurse to approach them with verbal and written information (see Trial Schema, Figure 1). If willing, a verbal discussion can take place that day, otherwise patients will be given a copy of the Patient Information sheet and then contacted by the trial nurse to discuss the trial a few days later. Most patients will attend the Pre-Operative Assessment Clinic (POAC) within 2 weeks of the decision to operate, and the trial nurse will meet them there to answer any further questions. If willing to participate, they will be consented and randomised, following baseline data collection, at that visit. Randomisation at this point will enable workforce planning for the day of surgery, as well as participant education on the ERP and trial data collection. However, some patients may be initially identified and approached at the POAC stage, and may therefore be randomised up to and including the day of admission.

The trial interventions will take place in the anaesthetic room on the day of surgery, with intensive data collection up to the third day post-operatively, and further specific data collection up to 30 days post-operatively.

#### Sample size

The primary end point is difference in VAS pain scores on movement at 24 hours post-operatively. The Kelly study [51] showed that the minimum clinically significant VAS pain score when managing severe pain was 10 mm. Standard deviations (SD) varying from 14 mm to 18 mm have been reported [3–7], thus we have estimated a standard deviation of 18 mm for our study. To achieve 85% power to detect a 10 mm difference in the primary end point, from a VAS pain score on movement at 24 hours of 40 mm (SD =18 mm) in the TEA group, to 30 mm (SD =18 mm) in the RSC group, at the 5% level (two-sided *t*-test), will require 60 patients in each arm of the study.

An additional 6 subjects will be recruited in each study arm to cover a maximum of 10% losses (the MASTERS study [4] had only 3.5% losses), bringing the total sample to 132 subjects recruited over 24 months. This sample size is realistic as we have a recruitment pool of 130 admissions per year and expect 70% to be both eligible and willing to participate.

#### Recruitment

Relevant surgical teams, and in particular the specialist nurses, have been educated on trial eligibility criteria and will communicate clinic dates for potential participants to the trial nurse once a decision to operate is made. Most of these decisions take place at the weekly Multidisciplinary Cancer Meeting, and the trial nurse will therefore attend the colorectal meetings.

In addition, all relevant surgical outpatient clinics have been provided with eligibility posters and trial nurse contact details as further reminders. POAC personnel have also received this material, as have the admissions clerks who are responsible for booking patients into outpatient and operation dates. This will identify any additional patients who may have been missed initially. Clinic dates for benign disease will similarly be communicated to researchers (this only applies to colorectal surgery) for all potentially eligible patients.

The trial nurse will then attend the relevant surgical clinic where patients will be offered major surgery (or POAC if identified at this stage). Eligible patients will be asked by the surgical team if they would be willing to be approached about the study by the research team, or have their contact details forwarded to the research team. If they agree, the research nurse will be on hand for an initial meeting where a verbal explanation of the study will be provided, along with a copy of the Patient Information Sheet.

Some patients may not feel ready to have a further research related discussion on the same day, but still be open to the idea of participation. The research nurse will gain their permission to contact them in a few days to discuss the study, and they will be provided with the Patient Information Sheet and contact details. As part of their surgical pathway, patients will be given an appointment at the POAC within 2 weeks. The research nurse will meet them again at this clinic and ascertain if they wish to participate: if so, they will be randomised, following consent and collection of baseline data. Patients who are approached for the first time at the POAC may have this second discussion (± randomization) on the day of admission, allowing time for consideration.

Only patients with mental capacity and the ability to consent will be recruited. Patients who are eligible but not randomised, and those who fulfil the inclusion criteria but meet one or more of the exclusion criteria, will be recorded in the TERSC Screening Log, irrespective of whether consent is obtained.

All stakeholders, that is anaesthetists, surgeons and specialist nurses, will be updated regularly of recruitment to the trial to ensure continued support and encouragement with positive feedback. The monthly Trial Management Group and the 6-monthly Trial Steering Committee (TSC) will scrutinise the recruitment rate and provide further suggestions and recommendations to increase any failing recruitment.

## Methods: assignment of interventions

### Allocation: sequence generation

Patients will be stratified by operation type and age, with three surgical strata (radical cystectomies, major colonic excisions and major rectal excisions) and two age strata (up to and above forty years of age) by computer generated random numbers. Of the 66 patients recruited into each arm of the study, we expect 50% to be major colonic excisions, 30% to be major rectal excisions and 20% to be radical cystectomy cases. Because we expect to see substantially more patients in the over-40 group, a 2:1 inclusion rate between the age groups is expected. It should be noted that the radical cystectomy group will only contain patients over the age of forty, and, therefore, in total there will only be five strata. The Trial Statistician has provided the InForm database designers with lists of the random assignment blocks, without any involvement of the CI or trial nurse.

### Allocation: concealment mechanism

The allocation is provided electronically by the InForm system as described above and is thus concealed to the research team.

### Implementation

The trial nurse will enrol patients, and once eligibility criteria, operation type and demographics are entered, the InForm system will electronically randomise participants and assign the interventions.

### Blinding

Only the data analysts (trial statistician) will be blinded to the assigned intervention when assessing the primary outcome measure.

## Methods: data collection, management and analysis

### Data collection methods

The trial research nurse is responsible for recruitment and data collection and has been trained by the CI. This training included the correct collection of all trial data along with definitions of data items and clinical scoring tools, correct assessment of pain intensity and other VAS as well as the PQRS score.

Baseline data is obtained in the POAC directly from the patient and the case notes. All outcome data is either obtained from the Patient Diaries, case notes, prescription, fluid balance and observation charts, or directly by the research nurse: for example, timing of intervention insertion, duration of surgery, length of wound, presence and location of drains and stomas, the use of cardiac output monitoring to optimise fluid management and haemodynamic data from monitors.

Patients are educated in the completion of the Patient Diaries by the research nurse at the pre-operative assessment visit. The research nurse and clinical staff then prompt patients to self-complete the diaries at specified time points throughout their stay. These diaries capture the outcome measures regarding pain control, nausea, sleep quality, gut function and fitness for discharge.

There are no paper Case Report Forms and all data collected by researchers will be entered directly onto a trial-specific eCRF (electronic Case Report Form) on the InForm database. This system promotes data quality by preventing duplicate data entry, generating queries whenever data deviates from set ranges, and requiring completion of mandatory fields as defined by the CI. An audit trail is generated for any data queries, which must be resolved before sign-off of completed data by the CI.

A local Research and Development Department quality manager will audit the quality of data collection periodically and the Data Management and Ethics Committee (DMEC) will forward reports of data quality from InForm to the TSC. Full data collection will continue for all patients that deviate from the protocol, and patients who discontinue in the trial will still be assessed for surgical complications, hospital length of stay and adverse events up to 30 days.

Several validated scoring systems are used for predicting risk and for various outcome metrics. Peri-operative risk will be measured using the ASA (American Society of Anesthesiologists) score [52], and the Physiologic and Operative Severity Score for the enUmeration of Mortality and morbidity (p-POSSUM) score (a validated general surgical risk prediction score) [53]. Quality of Recovery will be assessed using the PQRS. This is a rapidly conducted instrument allowing assessment of several aspects of recovery over time. Physiological, nociceptive, emotional and cognitive domains are assessed, with a baseline (pre-operative) measurement followed by measurements over several time points. A feasibility study demonstrated high face validity with applicability to a wide age range, diverse languages, cultures, and physical abilities [44].

Details regarding the research interventions, for example, insertion, maintenance, protocol violations, adverse events, compliance with protocolised adjuvant analgesia and the ERP pathway, and all additional opiate analgesia will be obtained from case notes or directly by the research nurse. The hospital electronic administration system will be interrogated at 30 days for any readmissions to hospital, ensuring full capture of any adverse events in this time. The patients will be contacted at 30 days to obtain a final PQRS recovery score by telephone, and this will be deemed the endpoint for the primary study. A subset of patients will go on to be interviewed for the nested qualitative study.

### Data management

All patient trial data will be entered directly into the secure web-based InForm database eCRF using a tablet device. Patient diaries, PQRS paper questionnaires and the medical notes will serve as source data. The CI will be responsible for data collection, quality and recording, however the collection of data will be delegated to the trial research nurse. This has been recorded in the Delegation of Trial Duties Log and authorised by the CI.

All data entered into the eCRF will be anonymised with only initials and date of birth entered. A separate and secure log will be kept with consent forms and identifiers to allow patient follow-up to 30 days. This is detailed in the Patient Information Sheet and emphasised on the consent form. During the conduct of the trial, all electronic patient data will be encrypted and all trial documents stored securely. On completion of the trial, all patient data (electronic and paper) and other trial documents will be archived securely and retained for 5 years at RBH in the case of paper documents and Imperial Clinical Trials Unit (CTU) in the case of electronic data.

Imperial CTU is registered under the Data Protection Act 1998 and all Imperial CTU staff have undergone data protection and International Conference on Harmonisation (ICH) Good Clinical Practice (GCP) training. The CI and all research staff at the Trial Centre have undergone GCP training.

The InForm database generates automatic alerts for missing data, invalid data, data duplication or data not conforming to the rules established for that data type. Thus, validation is ongoing throughout the data entry process. Final data validation occurs at Imperial CTU. This will ensure all data is complete, accurate and consistent. The trial nurse will resolve data queries generated by the database as soon as possible.

TERSC will be managed according to the Medical Research Council’s (MRC) Guidelines for Good Research Practice, Guidelines for Good Clinical Practice (GCP) in Clinical Trials, and Procedure for Inquiring into Allegations of Scientific Misconduct.

### Statistical methods

A full statistical analysis plan will be written, *a priori*, before the investigators are unblinded to any trial outcomes by the trial statistician. All analyses will be performed according to the intention-to-treat principle. An additional per protocol analysis will be performed and reasons for any protocol violations reported. Baseline covariates will be reported for the two groups and described using summary statistics. To determine if the two groups are balanced, standard hypothesis tests (*t*-test, Mann-Whitney *U*-test and Chi-squared test) will be applied. Prior to writing the statistical analysis plan, we will determine if the analyses of the primary or secondary outcomes need to be adjusted for significant clinical or demographic covariates. However, we will only adjust the analysis if significant differences in these covariates exist between the treatment and control groups. The primary analysis will test the null hypothesis that there is no difference in mean VAS pain scores on movement at 24 hours post-operatively between those receiving TEA and RSC analgesia. The independent sample *t*-test or Mann-Whitney *U*-test will be used to assess between group differences unless the preliminary analysis indicates a significant difference in the clinical or demographic covariates thought to have a significant impact on the primary outcome. If so, then analysis of covariance will be used to assess the primary outcome. As there are six follow-up measurements, a repeated measures analysis of variance will also be used to assess differences between groups, and over time.

The secondary outcomes will be analysed in a similar manner to the primary outcomes. Time to event variables, such as time to recovery of gut function and length of hospital stay will be analysed using Mann-Whitney *U*-test, log-rank or Cox regression if censoring occurs. To avoid missing data we will monitor the data on a regular basis, monthly, and when possible return to the source of the missing data to determine if data is available. At database lock, we will assess the nature of any missing data and use appropriate imputation methods to estimate missing values.

## Methods: monitoring

### Data monitoring

The DMEC will consist of an independent academic clinician and an independent statistician, and will meet every 6 months. Operating under the DAMOCLES Charter [54, 55], the DMEC will receive reports on recruitment rates, adverse events, completeness of data collection and protocol violations, and will report in turn to the TSC.

### Harms

Adverse events (AE) and serious adverse events (SAE) are defined in this trial according to Directive 2001/20/EC, 4 April 2001, of the European Parliament (Clinical Trials Directive) and ICH (International Conference on Harmonisation) GCP E6 guidelines. All AE occurring in the 30-day follow-up period will be recorded and evaluated for severity and causality. Severity will be graded 1 (mild) through 5 (fatal) and causality will be graded as either none, unlikely, possible, probable or definitely related to the intervention. SAE will also be evaluated for expectedness. SAE will be reported to the Chief Investigator (CI) within 24 hours. The CI will assess all SAE to determine the need for expedited reporting to the DMEC, TSC and Research Ethics Committee.

### Auditing

The hospital Research and Development Department quality manager will conduct local monitoring of trial quality after the first patients have been enrolled. The trial database provides on-going data quality checking. The DMEC will look at recruitment rate, data quality and adverse event reporting and provide a summary report every 6 months.

## Ethics and dissemination

### Research ethics approval

The Greater Manchester East Research Ethics Committee granted ethical approval (REC reference: 13/NW/0782 61767) on the 19 November 2013. Informed patient consent will be obtained from all participants before enrolment in the trial.

### Protocol amendments

Any significant protocol amendments will be communicated to the Research Ethics Committee and tabulated along with the amendment date and updated version number in the protocol. The local research team, the Trial Management Group, Trial Steering Committee and the Data Monitoring and Ethics Committees will all be informed.

The National Institute for Health Research (NIHR) portfolio team and the Controlled Trials Registry where the trial is registered will also be informed.

### Consent

Please refer to the ‘qualitative study’, ‘participant timeline’, and ‘recruitment’ sections above for details on consent.

### Confidentiality

Please refer to the ‘data management’ section above for details on confidentiality.

### Declaration of interests

None.

### Access to data

The trial statistician and the DMEC will have access to the final trial dataset.

### End of trial

This will occur when the final patient has completed their 30-day post-operative follow-up and the final qualitative interview has taken place. At this point, all electronic and paper trial data will be securely archived for a minimum of 5 years in accordance with ICH GCP guidelines. Arrangements for confidential destruction of all documents will then be made. The trial may be stopped early upon recommendation of either the TSC or DMEC. If this occurs, all randomised patients will continue to be followed up as per the trial protocol.

### Post-trial care

Due to the short duration of the intervention no post-trial care is required.

### Dissemination policy

Following completion of the trial a report will be produced for the NIHR who have funded the trial. Several peer reviewed journal publications will be produced reporting various aspects of the trial and results will be presented at relevant scientific meetings. A lay version of the results will be available for all trial participants who request a copy, and for all hospital staff.

## Discussion

Epidurals are in routine use in the National Health Service (NHS) [2, 18], and their risks and benefits are well described; therefore this study does not confer any additional risk to trial patients. RSC carry a theoretical risk of needle damage to viscera on insertion (very unlikely under ultrasound guidance) and local anaesthetic toxicity (this applies to both interventions). However, to date no cases of either have been reported [23]. Clinical experience suggests good analgesia and easy mobility with RSC [38, 40] but the trial is required to provide direct comparison with epidurals.

We considered whether to blind the main study to minimise any potential bias. Unfortunately, blinding of staff would be impossible, as they are responsible for administering the drugs (by very distinct routes, with different pumps and different settings), as well as monitoring for adverse events related to the techniques. Single blinding (patient) was then considered but this was rejected for two reasons. These were the high likelihood of accidental unblinding, and the unacceptability of inserting sham epidurals or RSC. Allocation concealment will avoid selection bias but observer bias remains a risk. Blinding the data analyst to the intervention group will ameliorate this.

As we will be studying three different categories of surgery, with different expected rates of morbidity and hospital stay (secondary outcome measures), we have stratified randomisation to account for this and ensure these categories are balanced. The same applies to age. The vast majority of cancer cases are older-aged, however a younger cohort of patients with inflammatory bowel disease exists, for whom providing pain relief is notoriously difficult and hospital stays are often longer.

VAS pain scores are a necessary primary outcome measure for pain studies as they allow quantitative statistics. They are efficient to collect and easy for patients to understand, but are recognised as subjective in nature with individual variation, error in administration and limited to measuring pain intensity. Therefore, we will also be comparing a host of other measurements of quality of analgesia: for example, time to first rescue opiate, the amount of additional opiate, measurements of functional mobility and breathing, overall satisfaction with pain relief, measures of recovery and patient experience with their intervention. Several important secondary outcomes will allow further differentiation between the interventions regarding safety, experience and functional recovery.

There are several unique pragmatic aspects to the study design which will help to reduce confounders and improve the generalisability of results. Firstly, patients will have all other aspects of care standardised by inclusion in the ERP, with the respective trial interventions the only difference in processes of care. It is important to note that the respective trial interventions are not merely the insertion of an epidural compared to the insertion of a RSC, but rather two overall packages of analgesia and the way in which the interventions are managed: for example, the use of transdermal fentanyl patches in both groups at different appropriate time points, the universal use of gabapentin and simple analgesia, the portable pump system delivering the local anaesthetic via the RSC, robust protocols to optimise inadequate epidural or rectus sheath blocks and any breakthrough pain, and patient education pre-operatively regarding their allocated intervention, their role in their recovery and diary completion. A clear protocol states when an intervention is considered to have failed and the conversion to rescue analgesia.

Secondly, the use of transdermal fentanyl patches deserves further mention. These are used routinely in our institution as a step down from epidural analgesia. In order to manage the visceral pain component in the RSC arm, the patches are applied, following a single dose of morphine and a further RSC bolus, in the final 45 minutes of surgery. Visceral pain, although severe, is short-lived (6 to 24 hours) [23] and in our experience is effectively managed with a 12 mcg or 25 mcg patch. Previous studies have used morphine PCA in addition to the RSC for this visceral pain. However, there is a danger that the RSC are not optimised in these cases, with morphine being relied upon for any breakthrough pain. This would negate the benefits of a potentially good block, and could result in the side effects of high dose opiates as previously discussed. A morphine PCA would also require an additional pump and would further complicate mobilisation. Instead, we make use of oral morphine for any opiate required in addition to the patch.

Aside from increasing the quality of care provided to all patients participating in this study, further benefits to our institution include acquisition of new clinical skills for anaesthetic and ward staff regarding the insertion and management of RSC, new research skills and development of a research ethos for all staff involved, and potential reduction in morbidity and consequently hospital stay, leading to economic benefit.

Many surgical procedures that have previously been performed via midline incision are now being performed laparoscopically [56] or via transverse incisions. Neither of these approaches requires the interventions described in this trial. However, emergency laparotomies will continue to require an open midline approach. This group has a mortality rate 5- to 10-fold greater than the elective population, comprising in excess of 5,000 cases per year due to bowel cancer alone in England [56]. These vulnerable patients are frequently managed with high dose systemic opiate as many have contraindications to epidural analgesia. Therefore, if RSC demonstrate benefit, the technique may have widespread application in this surgical cohort.

## Trial status

Recruitment began on the 10^th^ February 2014 with planned completion on the 10^th^ February 2016.

## Authors’ information

AK is a practicing Consultant in Anaesthesia and Intensive Care Medicine, Clinical Lead for all Enhanced Recovery Programmes for Surgery, Director of Research and Innovation for his institution, and Specialty Lead for Anaesthesia and Perioperative Medicine for the Greater Manchester Clinical Research Network as part of the National Institute for Health Research (NIHR), UK.

ACG is Clinical Senior Lecturer in Intensive Care Medicine at Imperial College London and is an NIHR Clinician Scientist award holder. He is also Deputy Director of Research of the Intensive Care Foundation.

SGB is a lecturer with research interests which include patients’ experiences of cancer (symptoms, care and services; using qualitative approaches to gather experiential data, and RCTs), physical and practical problems experienced by cancer survivors and the development, validation and use of patient reported outcome measures (PROMs).

## Electronic supplementary material

Additional file 1: **Rectus sheath catheter insertion technique.** (DOCX 16 KB)

## Abbreviations

AE: adverse event
ASA: American Society of Anesthesiologists score
CI: chief investigator
CTU: Clinical Trials Unit
eCRF: electronic Case Report Form
DMEC: Data Management and Ethics Committee
ERP: Enhanced Recovery Programme
GCP: Good Clinical Practice
GI: gastrointestinal
MAC: minimum alveolar concentration
NHS: National Health Service
NIHR: National Institute for Health Research
PCA: patient controlled analgesia
POAC: Pre-operative Assessment Clinic
POMS: Post-Operative Morbidity Survey
p-POSSUM: Physiologic and Operative Severity Score for the enUmeration of Mortality and morbidity
PQRS: Post-operative Quality Recovery Scale
R&D: research and development
RBH: Royal Blackburn Hospital
RCT: randomised controlled trial
RfPB: Research for Patient Benefit programme
RSC: rectus sheath catheters
SAE: serious adverse event
TEA: thoracic epidural analgesia
TERSC: Thoracic Epidural analgesia versus Rectus Sheath Catheter study
TSC: Trial Steering Committee
VAS: Visual Analogue.
