# Supplementary material for: Thoracic Epidural analgesia versus Rectus Sheath Catheters for open midline incisions in major abdominal surgery within an enhanced recovery programme (TERSC): study protocol for a randomised controlled trial
Source: Trials. 2014 Oct 21;15:400. doi: 10.1186/1745-6215-15-400 (PMC4223757; doi:10.1186/1745-6215-15-400)
Supplement: Supplementary file 1 — Additional file 1: Rectus sheath catheter insertion technique. (DOCX 16 KB) [file 13063_2014_2276_MOESM1_ESM.docx]

**Additional file 1**

**Rectus Sheath Catheter Insertion Technique (insertion video by the author available at https://www.youtube.com/watch?v=6gHSlxCIH28)**

- Immediately after induction of general anaesthesia.
- Aseptic technique using large sterile drape with large aperture for adequate ultrasound access to the abdomen.
- Under direct ultrasound guidance using the Sonosite Micromaxx^TM^, Sonosite Titan^TM^ or Sonosite S-Nerve portable ultrasound machine with a sheathed linear transducer.
- Linea alba visualised in the upper abdomen.
- Probe then moved laterally to visualise the main body of the rectus abdominis muscle.
- The posterior rectus sheath and transversalis fascia are clearly visible as a set of ‘tramlines’ posterior to the rectus muscle.
- The ventral rami of the intercostal nerves supplying the anterior abdominal wall lie in this potential space between the posterior aspect of the rectus muscle and the ‘tramlines’.
- The ultrasound probe is then rotated into a coronal plane.
- An in-plane approach is used to insert a 16G Tuohy needle (Pajunk Rectus Sheath set 0721153-49).
- Advance the Tuohy needle until the tip lies on top of, or just anterior to the ‘tramlines’ but posterior to the rectus muscle.
- When the tip reaches the posterior sheath (tramlines) the correct position is confirmed by injecting a bolus of normal saline to separate the planes and achieve hydro-dissection.
- A further 20ml of 0.25% bupivacaine is injected down the Tuohy needle and this further opens up the potential space between the rectus muscle and posterior rectus sheath, allowing the epidural catheters to be inserted.
- The hydro-dissected space and catheters are easily visible on ultrasound confirming their correct positioning, and must be visualised before accepting the catheter position.
- The Tuohy needle is removed and the catheter is tunnelled subcutaneously in a cephalad direction to above the xiphisternum keeping it clear of the surgical field.
- The procedure is repeated on the opposite side and both catheters are secured with the dedicated fixation dressing provided in the Pajunk Rectus Sheath Catheter set.
